# The complete chloroplast genome of *Pertya phylicoides* (Asteraceae, Pertyeae): a shurby endemic species from China

**DOI:** 10.1080/23802359.2020.1722763

**Published:** 2020-01-31

**Authors:** Bin Wang, Qing Zhao, Xiu-Hua Wang, Zhi-Xi Fu

**Affiliations:** a School of Mathematical Sciences, Sichuan Normal University, Chengdu, PR China;; b Chengdu City Construction and Development Research Institute, Chengdu, PR China;; c State Key Laboratory of Systematic and Evolutionary Botany, Institute of Botany, the Chinese Academy of Sciences, Beijing, PR China;; d University of Chinese Academy of Science, Beijing, PR China;; e College of Life Sciences, Sichuan Normal University, Chengdu, PR China

**Keywords:** Asteraceae, *Pertya phylicoides*, chloroplast genome, endemic species

## Abstract

This study was the first report about complete chloroplast genome of *Pertya phylicoides* (Asteraceae: Pertyeae), a critically shurby plant. The complete chloroplast genome of *Pertya phylicoides* was 153,379 bp in length and a typical circular structure, which comprises a pair of inverted repeat (IR) region of 25,191 bp divided by a large single-copy (LSC) region of 84,535 bp and a small single-copy (SSC) region of 18,462 bp. The general GC content was 37.6% in the whole sequence, whereas corresponding values of 35.6%, 31.2%, and 43.1% in the LSC, SSC, and IR regions, respectively. The whole genome contained 113 unique genes, including 79 protein-coding genes, 4 ribosomal RNA genes, and 30 tRNA genes. The phylogenetic hypotheses obtained based on the analyses of 21 cp genomes indicates *Pertya phylicoides* was supported as a sister group to the tribe Carduoideae.

The *Pertya* Sch. Bip. is the shrub genus (17 species) mainly distributed in China (16 endemic species), with a few species in adjacent Japan, Thailand, Pakistan, and Afghanistan (Gao et al. 2011). The shrub *Pertya phylicoides* Jeffrey is a Chinese endemic species distributed in the dry valleys of Xizang, Yunnan, and Sichuan province. It also has ecological value. So far, the complete chloroplast genome of *Pertya phylicoides* has not yet been published. A good knowledge of genomic information of this species would contribute to the formulation of protection strategy and the study of genome diversity and species diversity. In this study, we assembled and characterized its complete chloroplast genome (Gen-Bank accession no. MN935435) from Illumina sequencing data.

Fresh leaves of *Pertya phylicoides* were collected from Muli, Sichuan Province, China, and deposited in the Herbarium of Sichuan Normal University, SCNU (specimen no.: Z.X. Fu 4038). High-quality total genomic DNA was extracted from *ca*. 6 cm^2^ sections of the silica-dried leaf using improved Tiangen Plant Genomic DNA Kits, then 4 μl RNAseA and 20 μl Proteinase K were added after incubation (65 °C). Total DNA was directly constructed using short-insert of 150 bp length libraries and sequenced on the Illumina Genome Analyzer (Hiseq 2000) according to the manufacturer’s protocol (Illumina, San Diego, CA, USA) by ORI-GENE, Beijing. Generally, more than 6 Gb of data were obtained for complete cp genome of *P. phylicoides*. Data were *de novo* assembled in CLC Genomic Workbench v11 (CLC Bio, Aarhus, Denmark) and the consensus were sequenced in Geneious R11.1.5 (Biomatters Ltd., Auckland, New Zealand) with the referenced chloroplast genome sequence of *Saussurea polylepis* (Accession: MF695711). The chloroplast genome was annotated using a web-based annotation program GeSeq (https://chlorobox.mpimp-golm.mpg.de/geseq.html), edited manually, and imaging was done with OGDraw v1.2 (Lohse et al. 2013). The complete chloroplast genome of *P. phylicoides* was 153,379 bp (GenBank Accession No. MN935435) in length with a typical circular structure, comprising a pair of inverted repeat (IR) of 25,191 bp divided by a large single-copy (LSC) region of 84,535 bp and a small single-copy (SSC) region of 18,462 bp. The general GC content was 37.6% in the whole sequence, with corresponding values of 35.6%, 31.2%, and 43.1% in the LSC, SSC, and IR regions, respectively. The whole genome contained 113 unique genes, including 79 protein-coding genes, 4 ribosomal RNA genes, and 30 tRNA genes.

To construct the phylogenetic tree, all of the cp genome sequences were aligned in MAFFT (Katoh and Standley 2013). A maximum likelihood analysis based on the GTRGAMMA model was performed with RaxML v7.2.8 on the CIPRES (Stamatakis et al. 2008; Miller et al. 2010) using 1000 bootstrap replicates with *Kalopanax septemlobus* (Thunb.) Koidz. (Araliaceae) and *Anthriscus cerefolium* (L.) Hoffm (Umbelliferae) as the outgroup. Based on limited materials, the phylogenetic hypotheses obtained with the analyses of 21 cp genomes indicates *P. phylicoides* was supported as a sister group to the tribe Carduoideae (Bootstrap support = 100, Figure 1). The complete plastome sequence of *P. phylicoides* will provide a useful resource for the conservation genetics of this species as well as for the phylogenetic studies for Asteraceae.

**Figure 1.**
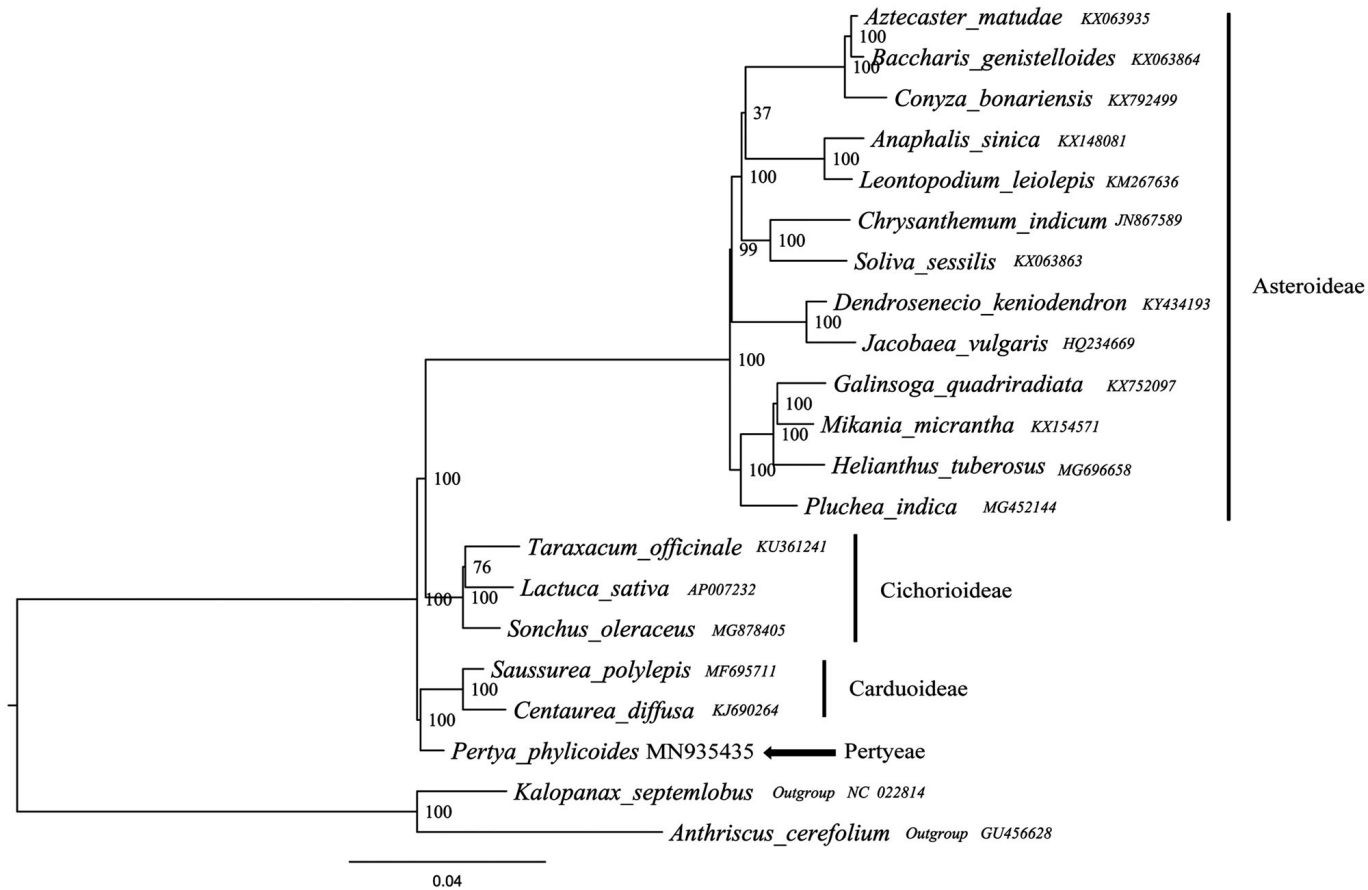
The best Maximum likelihood (ML) phylogram inferred from 21 chloroplast genomes in Asteraceae (bootstrap value are indicated on the branches).
